# Prediction of Sustained Virological Response to Telaprevir-Based Triple Therapy Using Viral Response within 2 Weeks

**DOI:** 10.1155/2014/748935

**Published:** 2014-09-28

**Authors:** Hideyuki Tamai, Ryo Shimizu, Naoki Shingaki, Yoshiyuki Mori, Shuya Maeshima, Junya Nuta, Yoshimasa Maeda, Kosaku Moribata, Yosuke Muraki, Hisanobu Deguchi, Izumi Inoue, Takao Maekita, Mikitaka Iguchi, Jun Kato, Masao Ichinose

**Affiliations:** Second Department of Internal Medicine, Wakayama Medical University, 811-1 Kimiidera, Wakayama, Wakayama Prefecture 641-0012, Japan

## Abstract

The aim of the present study was to predict sustained virological response (SVR) to telaprevir with pegylated interferon (PEG-IFN) and ribavirin using viral response within 2 weeks after therapy initiation. Thirty-six patients with genotype 1 hepatitis C virus (HCV) and high viral load were treated by telaprevir-based triple therapy. SVR was achieved in 72% (26/36) of patients. Significant differences between the SVR group and non-SVR group were noted regarding response to prior PEG-IFN plus ribavirin, interleukin (IL)28B polymorphism, amino acid substitution at core 70, cirrhosis, hyaluronic acid level, and HCV-RNA reduction within 2 weeks. Setting 4.56 logIU/mL as the cut-off value for HCV-RNA reduction at 2 weeks, the sensitivity, specificity, positive predictive value, negative predictive value, and accuracy for predicting SVR were 77%, 86%, 95%, 50%, and 79%, respectively, and for neither the IL28B minor allele nor core 70 mutant were 80%, 71%, 91%, 50%, and 78%, respectively. In conclusion, evaluation of viral reduction at 2 weeks or the combination of IL28B polymorphism and amino acid substitution at core 70 are useful for predicting SVR to telaprevir with PEG-IFN and ribavirin therapy.

## 1. Introduction

To date, although pegylated interferon (PEG-IFN) and ribavirin combination therapy has been standard care for patients with hepatitis C virus (HCV) infection, the sustained virological response (SVR) rate in patients with genotype 1 and high viral load is only approximately 40–50% [1]. Recently, some direct antiviral agents (DAAs) against HCV have been developed. The first-generation nonstructural 3/4A protease inhibitor, telaprevir, became available for clinical use in November 2011 in Japan. Triple therapy with telaprevir, PEG-IFN, and ribavirin has significantly improved SVR rates to around 70% [2]. However, compared with PEG-IFN plus ribavirin therapy, triple therapy can produce some severe adverse reactions, including serious skin disorders, exacerbation of anemia, and renal dysfunction [3–5]. Furthermore, the safety of telaprevir-based triple therapy in elderly or cirrhotic patients has not been established. However, as elderly and/or cirrhotic patients are at high risk of developing hepatocellular carcinoma, antiviral therapy should be commenced as soon as possible for these patients [1]. Therefore, the prediction of efficacy and assessment of tolerability for each individual who might receive telaprevir-based triple therapy are crucial for deciding the optimal treatment strategy. If therapeutic efficacy can be accurately predicted before or in the very early stages of treatment, risky and unnecessary treatment can be avoided.

Regarding pretreatment predictors for efficacy in telaprevir-based triple therapy, interleukin (IL)28B single nucleotide polymorphism (SNP), amino acid substitution at core 70, and response to previous therapy have been reported as useful predictors [6–8]. The Japanese guidelines for the management of hepatitis C virus infection [1] recommend that analysis of IL28B SNP and amino acid substitution at core 70 should be performed to enable selection of the optimum therapy regimen; telaprevir-based triple therapy is not recommended in patients with both the IL28B minor allele and mutant type of amino acid substitution at core 70. However, neither the test for the IL28B SNP nor the test for amino acid substitution at core 70 has been approved by the Japan National Medical Insurance System.

Extended rapid virological response (RVR), defined as serum HCV-RNA undetectable at both treatment week 4 and week 12, is also known to be one of the significant predictors of SVR to telaprevir-based triple therapy [9, 10]. However, the best time point has not been elucidated for predicting SVR using viral response. The aim of the present study was to evaluate whether SVR to telaprevir-based triple therapy can be more accurately predicted on the basis of super rapid virological response within 2 weeks of therapy initiation than RVR and whether the predictability for SVR by virological response within 2 weeks is comparable with that by the combination of IL28B SNP and amino acid substitution at core 70.

## 2. Methods

### 2.1. Patients

A total of 36 consecutive patients with genotype 1 high viral load (more than 5.0 logIU/mL) were enrolled from March 2012 to June 2013 in our hospital. Exclusion criteria were (1) pregnant women, women who may have been pregnant, lactating women, men whose partners were pregnant, or men whose partners hoped to become pregnant; (2) patients who used shosaikoto (a Kampo medicine); (3) intractable heart disease; (4) renal failure or renal dysfunction with creatinine clearance <50 mL/min; (5) patients with uncontrollable psychoneurotic disorders; (6) hemoglobin (Hb) level <11 g/dL; (7) platelet count <60,000/mm^3^; (8) white blood cell count <1500/mm^3^ (or granulocyte count <1000/mm^3^); and (9) hepatic failure or all types of cancer. The potential benefits and risks of the present study were explained to all patients before obtaining written informed consent. The protocol was approved by the Ethics Committee of Wakayama Medical University (no. 1081) and conformed to the Helsinki Declaration.

### 2.2. Treatment Regimens

Telaprevir (Telavic; Mitsubishi Tanabe Pharma, Osaka Japan), PEG-IFN-alpha-2b (Peg-Intron; MSD, Tokyo, Japan), and ribavirin (Rebetol; MSD) were used. PEG-IFN-alpha-2b at 1.5 *μ*g/kg was administered subcutaneously once per week for 24 weeks; ribavirin was given orally for 24 weeks (1000 mg/day for patients weighing more than 80 kg, 800 mg/day for patients weighing between 80 and 60 kg, and 600 mg/day for patients weighing less than 60 kg); and telaprevir was given orally at a dose of 750 mg every 8 hours after meals for 12 weeks from the start of therapy.

However, in a clinical trial of telaprevir-based triple therapy in Japan, elderly patients or patients with cytopenia due to cirrhosis were not included, and grade 3 anemia occurred more frequently in the telaprevir group than in the group who did not receive telaprevir [2]. The Japanese guidelines for the management of HCV infection recommend reduced doses of the combination therapy of PEG-IFN and ribavirin for compensated cirrhosis [1]. A pilot study of telaprevir-based triple therapy for elderly patients suggested that triple therapy with telaprevir 1500 mg seems safe and efficacious for elderly Japanese patients [11]. In addition, Sezaki et al. [12] also reported that, compared with telaprevir 2250 mg, telaprevir 1500 mg/day was associated with lower rates of anemia and similar antiviral efficacy, especially among elderly Japanese patients. Therefore, for safety, reduced doses of telaprevir (1500 mg), PEG-IFN-alpha-2b (1.0 *μ*g/kg), and ribavirin (200 mg less than the recommended dose) were initially administered in the present study for patients with any of the following: (1) age ≥65 years; (2) white blood cell count <2000/mm^3^; (3) platelet count <130,000/mm^3^; (4) Hb level <13 g/dL; (5) comorbid disorder such as heart disease, cerebrovascular disease, thyroid disease, psychiatric disease, autoimmune disease, or uncontrolled diabetes; or (6) low body weight (<40 kg).

During treatment, reduction in the telaprevir dose was not permitted. The doses of PEG-IFN-alpha-2b and ribavirin were reduced or discontinued based on the following criteria. (1) If the Hb fell below 10 g/dL, the dose of ribavirin was reduced by 200 mg from the starting dose, and if the Hb fell below 8.5 g/dL, the ribavirin was discontinued; (2) if the granulocyte count fell below 500/mm^3^ or the platelet count fell below 30,000/mm^3^, the PEG-IFN was discontinued; and (3) if deemed necessary by the attending physician because of adverse events, the telaprevir, PEG-IFN, and ribavirin were all discontinued. The dose of PEG-IFN or ribavirin was increased back to the starting dose if the cytopenia improved. If there was no improvement in hematological parameters within 4 weeks, the therapy was discontinued. Although granulocyte colony-stimulating factor was used as supplementary treatment for granulocytopenia less than 500/mm^3^, erythropoietin was not used for anemia.

### 2.3. Laboratory Tests and Ultrasound

In all patients, laboratory tests and ultrasound examination were performed before therapy began. Fatty liver was defined as positive hepatorenal contrast on ultrasound B mode imaging. The amount of HCV-RNA was measured using quantitative real-time polymerase chain reaction (RT-PCR) (COBAS TaqMan HCV test version 1.0; Roche Diagnostics, Branchburg, NJ, USA). High viral load was defined as more than 5.0 logIU/mL using quantitative RT-PCR. HCV genotype was determined according to Simmonds' classification [13]. The amount of HCV-RNA (AccuGene m-HCV, Abbott Japan, Tokyo, Japan) and HCV core antigen levels (ARCHITECT HCV; Abbott Japan, Tokyo, Japan) were measured simultaneously at three time points: the day of therapy initiation and at weeks 1 and 2. Serum levels of hyaluronic acid and type IV collagen 7S were measured for the assessment of liver fibrosis on the day of therapy initiation. Amino acid 70 and 91 substitutions in the HCV core region [14] were also measured on the day of therapy initiation. At core 70, arginine was defined as the wild type and glutamine or histidine as mutant types. At core 91, leucine was defined as the wild type and methionine as the mutant type. During therapy, quantitative HCV-RNA (COBAS TaqMan HCV test; Roche Diagnostics), biochemical analyses including blood counts, serum alanine aminotransferase (ALT), and aspartate aminotransferase (AST), were performed every 4 weeks up to 24 weeks after the end of therapy. After treatment, the SNP of IL28B (rs8099917) that was reported as a pretreatment predictor for the efficacy of PEG-IFN plus ribavirin therapy in Japanese patients [15] was additionally evaluated, after obtaining written informed consent for genome analysis from each patient. Homozygosity for the major allele (T/T) was defined as the IL28B major type, and heterozygosity (T/G) or homozygosity for the minor allele (G/G) was defined as the IL28B minor type.

### 2.4. Assessment of Effectiveness

During IFN therapy, rapid virological response (RVR) was defined as undetectable HCV-RNA using quantitative RT-PCR (COBAS TaqMan PCR assay; Roche Diagnostics) at week 4 after therapy initiation. SVR was defined as follows: the HCV-RNA measured using the TaqMan PCR assay was negative at the end of therapy and remained negative for 24 weeks after the end of therapy. No response was defined as detectable HCV-RNA at week 24 from treatment initiation or at the end of treatment. Relapse was defined as HCV-RNA-negative at the end of therapy but positive at 24 weeks after the end of therapy. The viral response within two weeks after therapy initiation was assessed by viral level and viral reduction from baseline viral load at each time point.

### 2.5. Assessment of Safety and Tolerability

Patients were assessed for safety and tolerability during treatment by their attending physicians, who monitored adverse events and laboratory abnormalities, such as blood cell counts, every week up to week 12 and monthly thereafter. The incidence and reasons for therapy discontinuation due to adverse effects were analyzed.

### 2.6. Statistical Analysis

Adherence to each medication (telaprevir, PEG-IFN-alpha-2b, and ribavirin) was assessed separately and was calculated based on the recommended doses. Therapeutic effectiveness was evaluated using intention-to-treat (ITT) analysis. Predictive factors for SVR were analyzed using a per protocol (PP) analysis that excluded patients who had discontinued therapy due to adverse events within 4 weeks. The Mann-Whitney *U* test was used to analyze continuous variables. Fisher's exact test or the chi-square test was used to analyze categorical variables. Each optimal cut-off value for continuous variables of SVR-predicting factors was decided by the Youden index method on the basis of the receiver operating characteristic (ROC) curve. The SVR predictability of significant SVR-contributing factors was evaluated by measuring the area under the curve (AUC). The sensitivity, specificity, positive predictive value (PPV), negative predictive value (NPV) for SVR, and accuracy were calculated for week 1, week 2, and RVR. Values of *P* < 0.05 were considered significant. The statistical software used was SPSS Ver. 21.0J for Windows (SPSS, Inc., Tokyo, Japan).

## 3. Results

### 3.1. Patients' Baseline Characteristics

The patients' baseline characteristics are summarized in Table 1. The patients were composed of 19 males and 17 females. Their median age was 61.5 years and the range was 29 to 72 years; 24 (67%) patients were <65 years old, and 12 (33%) were ≥65 years old. Four (11%) patients had experienced no response to prior PEG-IFN and ribavirin therapy. Thirty (81%) patients were treated by the reduced dose regimen.

### 3.2. Safety and Tolerability

Of the 36 patients, therapy was discontinued in 3 (8%) patients due to adverse events. The discontinuation rate was 0% (0/24) in patients <65 years old and 25% (3/12) in patients ≥65 years old, showing significant difference between groups (*P* = 0.046). Eighteen (49%) patients were clinically diagnosed with liver cirrhosis using the morphologic appearance of cirrhosis with portal hypertension, as evidenced by portosystemic shunt or hypersplenism on imaging, laboratory tests, and/or liver histology. The discontinuation rate was 17% (3/18) in cirrhotic patients and 0% (0/18) in noncirrhotic patients, with no significant difference between groups (*P* = 0.229). The discontinuation rate was 33% (3/9) in elderly cirrhotic patients and 0% (0/27) in the other patients, showing significant difference between groups (*P* = 0.012). Reasons for therapy discontinuation were psychiatric disorder in 2 patients and severe nausea in 1 patient. All of the patients who required discontinuation of therapy stopped the therapy within 4 weeks after therapy initiation.

### 3.3. Therapeutic Efficacy

Overall, SVR was achieved in 72% (26/36) of the study patients, relapse occurred in 8% (3/36), and no response was observed in 19% (7/36) patients. In the 30 patients who received the reduced dose regimen, SVR was achieved in 67% (20/30) of the patients, relapse occurred in 10% (3/30), and no response was observed in 23% (7/30) patients. On the other hand, the SVR rate in the 6 patients who were able to use the recommended dose regimen was 100% (6/6). With respect to patient age, the SVR rate was 79% (19/24) in patients <65 years old and 58% (7/12) in patients ≥65 years old, showing no significant difference between groups (*P* = 0.247). The SVR rate was 50% (9/18) in cirrhotic patients and 94% (17/18) in noncirrhotic patients, showing no significant difference between groups (*P* = 0.072). However, the SVR rate was 44% (4/9) in elderly cirrhotic patients and 89% (24/27) in the other patients, showing significant difference between groups (*P* = 0.013).

### 3.4. Contributing Factors for SVR and Predictability of SVR

The results of univariate analysis of pretreatment factors contributing to SVR are shown in Table 2. Significant differences between the SVR group and the non-SVR group were noted regarding response to prior PEG-IFN plus ribavirin, IL28B SNP, core 70 amino acid substitution, and hyaluronic acid level. In addition, the results of univariate analysis of on-treatment factors contributing to SVR are shown in Table 3. Significant differences were noted in HCV-RNA level at week 4, RVR, and reduction of HCV-RNA within 4 weeks. However, no significant difference was noted in level and reduction of HCV core antigen at any time point, or adherence to treatment regimen. The comparison of significant predicting factors for SVR according to the AUC is shown in Table 4. The AUC of the reduction of HCV-RNA at 2 weeks was the highest. The sensitivity, specificity, PPV, NPV, and accuracy for predicting SVR according to the significant factors associated with SVR are summarized in Table 5.

### 3.5. Contributing Factors for Week 2 Response

Although it was considered that the independent factors contributing to SVR were analyzed by multivariate analysis, there were too many significant variables in view of the small numbers of patients. To validate whether the HCV-RNA reduction at week 2 was an independent factor associated with SVR, the pretreatment factors contributing to week 2 response were analyzed. The results of univariate analysis of pretreatment factors contributing to SVR are shown in Table 6. Response to prior PEG-IFN and ribavirin, cirrhosis, IL28B SNP, core 70 substitution, and hyaluronic acid level, which were significantly related to SVR, were not significant factors contributing to week 2 response. HCV-RNA level and core antigen were the only significant factors contributing to 2-week response. Therefore, week 2 response might be one of the independent factors contributing to SVR.

## 4. Discussion

Among patients with HCV infection, elderly cirrhotic patients have the highest priority for antiviral treatment because they are at the highest risk of liver-related death such as hepatic failure or hepatocellular carcinoma. Regarding the safety of triple therapy for cirrhotic patients, in the ANRS CO20 CUPIC study [16], although the early discontinuation rate within 16 weeks was 11.7%, a high incidence of serious adverse events (40%), death (1.2%), severe complications (severe infection or hepatic decompensation; 6.4%), and difficult management of anemia (requiring erythropoietin and transfusion) were observed. Furthermore, in an open label expanded access program cohort for evaluating the safety and efficacy of telaprevir-based triple therapy in patients with advanced fibrosis or cirrhosis involving 16 nations worldwide [17], although the early discontinuation rate was 12%, the mortality of the cirrhotic patients was 0.7%. The discontinuation rates in both of these studies might be acceptable for safety and tolerability; however, in common with both studies, age and female gender were independent predictors of severe anemia. In addition, low platelet count (<100,000/mm^3^) and low serum albumin level (<35 g/dL) were related to death or severe complications in the CUPIC study. Therefore, it is recommended that patients with factors of both low platelet count and low serum albumin level should not be treated with triple therapy. The telaprevir discontinuation rate in the present study was low (8%) regardless of the inclusion of many elderly and/or cirrhotic patients who are at high risk for severe adverse effects of interferon-based therapy. However, we considered that the reason for the low discontinuation rate was the reduced dose regimen.

Regarding the reduced dose of telaprevir, Sezaki et al. [12] reported a case control study for Japanese patients that compared patients who received telaprevir at a dose of 2250 mg/day and a dose of 1500 mg/day. These investigators reported that the discontinuation rate of patients receiving telaprevir 1500 mg/day was significantly lower than that of the patients receiving telaprevir 2250 mg/day (10% versus 25%), and that the SVR rates of both groups were equivalent (70% versus 83%). Therefore, these investigators suggested that the telaprevir-reduced regimen is safe and effective, especially for elderly and female Japanese patients. However, although in the present study, we reduced not only the telaprevir dose but also the PEG-IFN and ribavirin doses, the discontinuation rate of elderly and cirrhotic patients was high (33%) and significantly higher than that of the other patients. Furthermore, the SVR rate of the elderly and cirrhotic patients was low (44%) and significantly lower than in the other patients. Therefore, we suggest that even the reduced regimen should not be administered to elderly and cirrhotic patients. However, if the telaprevir-based triple therapy was to be applied for elderly cirrhotic patients, both tolerability and the prediction of efficacy must be strictly evaluated.

With respect to the prediction of the efficacy of telaprevir-based triple therapy, other investigators have already reported that amino acid substitution at core 70, IL28B SNP, cirrhosis, alpha-fetoprotein (AFP) level, and prior treatment response are significant pretreatment predictive factors of SVR [6, 18–20]. Regardless of the small number of patients in the present study, we found that prior treatment response, amino acid substitution at core 70, IL28B SNP, cirrhosis, and hyaluronic acid level were also significant predictive factors for SVR. Although the AFP level was not significant in the present study (*P* = 0.067), the AFP level would have been significant if the number of study patients had been much higher. Regarding on-treatment predictive factors, Shimada et al. [21] reported that RVR and very early viral response at week 1 were predictive factors; these investigators noted that the AUCs of the reduction in HCV-RNA levels at week 1 and of the IL28B minor allele were 0.754 and 0.777, respectively, and the predictability of both factors was equivalent. However, they did not evaluate the reduction in HCV-RNA levels at week 2. In the present study, comparison of AUC levels according to on-treatment factors within 4 weeks, including RVR, revealed that the best time point for predicting SVR using viral response was at week 2. Furthermore, the ability of HCV-RNA reduction at week 2 to predict SVR was the best among significant predictive factors, comparable to that of the combination of IL28B SNP and core amino acid substitution at core 70. Although the Japanese guidelines for the management of hepatitis C virus infection [1] do not recommend telaprevir-based triple therapy in patients having the IL28B minor allele and mutant type of amino acid substitution at core 70, neither of these tests have been approved by the national medical insurance system in Japan. Therefore, the evaluation of HCV-RNA reduction at week 2 may become an alternative to testing IL28B SNPs and core amino acid substitution. However, in order to validate the present results and to determine an optimal cut-off value, a further large-scale study is necessary.

In the present study, the amount of HCV-RNA was measured by the AccuGene method to evaluate virological response within 2 weeks, because the AccuGene method has a wider range and higher sensitivity than the TaqMan method. For the same reason, the amount of HCV core antigen (Ag) was measured by the ARCHITECT method of the third generation HCV core Ag test. In the present study, however, the best cut-off value for predicting SVR using HCV-RNA reduction at week 2 was set at 4.56 logIU/mL. Shimada et al. [21] also set the best cut-off value of predicting SVR using HCV-RNA reduction at week 1 at 4.7 logIU/mL. As the baseline high viral load is defined as more than 5.0 logIU/mL, and HCV-RNA reduction within 2 weeks can be evaluated within the range of either method, it is considered that the difference of sensitivity between the two methods is not significant for evaluating treatment response. Interestingly, although our previous reports indicated that the monitoring of core Ag within 2 weeks is useful for predicting SVR to PEG-IFN and ribavirin combination therapy [22, 23], core Ag was not useful for predictions in telaprevir-based triple therapy. This may indicate that telaprevir is one of the DAAs that strongly inhibit viral RNA replication.

Certain limitations must be considered when interpreting the results of the present study. The present study was small and included both naïve and previously treated patients. In addition, the *P* value of HCV RNA reduction at week 2 by assessing AUCs was almost similar to that of the various other significant predictors. There may be no significant difference among the AUCs of significant predictors for SVR. In order to validate the present results, further large-scale prospective studies are necessary.

In conclusion, from the analysis of predictors including viral dynamics, it was demonstrated that the best time point for predicting SVR to telaprevir with concomitant PEG-IFN and ribavirin therapy is at week 2, and that HCV-RNA reduction at 2 weeks is the most useful predictor among viral responses within 4 weeks after therapy initiation. As the predictability of HCV-RNA reduction at 2 weeks is equivalent to that of the combination of IL28B SNP and amino acid substitution at core 70, HCV-RNA reduction at week 2 has the potential to be used as an alternative predictor instead of the tests for IL28B SNP and amino acid substitution at core 70.

## Figures and Tables

**Table 1:** Baseline characteristics of the study patients.

	*n* = 36
Age (years)	61.5 (29–72)
Sex (male/female)	19/17
Height (cm)	159.3 (143.1–177.0)
Weight (kg)	62.1 (39.6–81.5)
BMI	24.2 (17.1–35.0)
Genotype (1a/1b)	1/35
Baseline HCV-RNA (LogIU/mL; TaqMan)	6.7 (4.8–7.5)
Response to prior PEG-IFN and ribavirin (NR/Relapse/Naive)	4/18/14
Fatty liver	6
Cirrhosis	18
Diabetes mellitus	7
IL28B, rs8099917 (TT/GT/GG)	24/11/1
Core 70 (wild/mutant/ND)	18/17/1
Core 91 (wild/mutant/ND)	26/9/1
WBC (/mm^3^)	4785 (2700–9300)
Hb (g/dL)	14.6 (11.6–18.6)
Platelets (/mm^3^)	16.5 (6.6–28.0)
ALT (IU/L)	46.0 (14–507)
*γ*-GT (IU/L)	35.5 (10–306)
Type VI collagen 7S (ng/mL)	4.4 (3.1–10.5)
Hyaluronic acid (ng/mL)	98.0 (12.3–839.0)
AFP (ng/mL)	4.5 (1.3–174.6)
Reduced dose regimen	30
Telaprevir adherence (%)	66.7 (0.6–100)
PEG-IFN adherence (%)	66.8 (5.5–100)
Ribavirin adherence (%)	66.7 (2.4–100)

Values are expressed as median (range). BMI, body mass index; HCV, hepatitis C virus; IL, interleukin; ND, not determined; WBC, white blood cells; Hb, hemoglobin; ALT, alanine aminotransferase; *γ*-GT, *γ*-glutamyl transferase; AFP, alpha-fetoprotein; PEG-IFN, pegylated interferon.

**Table 2:** Comparison of pretreatment factors between patients with and without sustained virological response.

Factors	SVR (*n* = 26)	Non-SVR (*n* = 7)	*P*
Age (years)	61	60	0.914
Sex (male/female)	13/13	5/2	0.413
Height (cm)	159.6	159.5	0.880
Weight (kg)	62.6	59.9	0.949
BMI	24.4	25.0	0.780
Baseline HCV-RNA (logIU/mL; TaqMan)	6.7	6.5	0.399
Baseline HCV-RNA (logIU/mL; AccuGene)	6.1	5.6	0.215
Baseline HCV core Ag (fmol/L)	4477.4	1524.4	0.330
No response to prior PEG-IFN and ribavirin	1	3	0.023
Fatty liver	6	0	0.301
Cirrhosis	9	6	0.030
Diabetes mellitus	4	1	1.000
IL28B (major/minor)	20/6	1/6	0.005
Core 70 (wild/mutant)	17/8	1/6	0.027
Core 91 (wild/mutant)	18/7	5/2	1.000
WBC (/mm3)	4995	4410	0.450
Hb (g/dL)	14.5	14.5	0.747
Platelets (/mm^3^)	16.9	12.2	0.099
ALT (IU/L)	49.5	43.0	0.714
*γ*-GT (IU/L)	33.5	49.0	0.199
Type VI collagen 7S (ng/mL)	4.4	8.1	0.054
Hyaluronic acid (ng/mL)	72.7	367.0	0.048
AFP (ng/mL)	4.1	11.2	0.067
Reduced dose regimen	20	7	0.301

Values are expressed as median. SVR, sustained virological response; BMI, body mass index; HCV, hepatitis C virus; Ag, antigen; IL, interleukin; WBC, white blood cells; Hb, hemoglobin; ALT, alanine aminotransferase; *γ*-GT, *γ*-glutamyl transferase; AFP, alpha-fetoprotein.

**Table 3:** Comparison of on-treatment factors between patients with and without sustained virological response.

Factors	SVR (*n* = 26)	Non-SVR (*n* = 7)	*P*
HCV-RNA level at week 1 (logIU/mL)	1.3	2.4	0.399
HCV-RNA level at week 2 (logIU/mL)	1.1	1.8	0.199
HCV-RNA level at week 4 (logIU/mL)	0	1.2	0.039
RVR	20	2	0.027
Reduction of HCV-RNA at week 1 (log)	4.5	3.4	0.003
Reduction of HCV-RNA at week 2 (log)	5.1	4.2	0.003
Reduction of HCV-RNA at week 4 (log)	6.6	5.4	0.010
HCV core Ag level at week 1 (fmol/L)	4.9	7.0	0.949
HCV core Ag level at week 2 (fmol/L)	0.8	2.4	0.399
Reduction of HCV core Ag at week 1	4476.5	1517.5	0.399
Reduction of HCV core Ag at week 2	4477.4	1524.1	0.330
Telaprevir adherence (%)	66.7	66.7	0.949
PEG-IFN adherence (%)	68.4	68.5	0.846
Ribavirin adherence (%)	70.9	75.0	0.682

Values are expressed as median. SVR, sustained virological response; HCV, hepatitis C virus; RVR, rapid virological response; Ag, antigen; PEG-IFN, pegylated interferon.

**Table 4:** Area under the receiver operating characteristic curve according to significant predicting factors for sustained virological response.

Factors	AUC	*P*	95% CI
Non-NR to prior PEG-IFN and ribavirin	0.695	0.118	0.441–0.949
IL28B major	0.813	0.012	0.632–0.994
Core 70 wild	0.769	0.032	0.577–0.960
Cirrhosis	0.755	0.041	0.563–0.948
Hyaluronic acid level	0.747	0.048	0.515–0.979
Reduction of HCV-RNA at week 1	0.849	0.005	0.682–1.000
Reduction of HCV-RNA at week 2	0.857	0.004	0.716–0.999
Reduction of HCV-RNA at week 4	0.813	0.012	0.663–0.963
RVR	0.742	0.053	0.524–0.905

AUC, area under the receiver operating characteristic curve; CI, confidence interval; NR, no response; PEG-IFN, pegylated interferon; IL, interleukin; HCV, hepatitis C virus; RVR, rapid virological response.

**Table 5:** Predictive values according to significant factors related to sustained virological response.

Significant predictive factors	Sensitivity	Specificity	PPV	NPV	Accuracy
Non-NR to prior PEG-IFN and ribavirin	96% (25/26)	43% (3/7)	86% (25/29)	75% (3/4)	85% (28/33)
IL28B major	77% (20/26)	86% (6/7)	95% (20/21)	50% (6/12)	79% (26/33)
Core 70 wild	60% (15/25)	86% (6/7)	94% (15/16)	38% (6/16)	66% (21/32)
No cirrhosis	65% (17/26)	86% (6/7)	94% (17/18)	40% (6/15)	70% (23/33)
Hyaluronic acid level (<346 ng/mL)	92% (24/26)	57% (4/7)	89% (24/27)	67% (4/6)	85% (28/33)
Reduction of HCV-RNA at week 1 (>4.36 logIU/mL)	73% (19/26)	86% (6/7)	95% (19/20)	46% (6/13)	76% (25/33)
Reduction of HCV-RNA at week 2 (>4.56 logIU/mL)	77% (20/26)	86% (6/7)	95% (20/21)	50% (6/12)	79% (26/33)
Reduction of HCV-RNA at week 4 (>6.40 logIU/mL)	65% (17/26)	100% (7/7)	100% (17/17)	44% (7/16)	73% (24/33)
RVR	77% (20/26)	71% (5/7)	91% (20/22)	45% (5/11)	76% (25/33)
Neither the IL28B minor nor core 70 mutant	80% (20/25)	71% (5/7)	91% (20/22)	50% (5/10)	78% (25/32)

PPV, positive predictive value; NPV, negative predictive value; NR, no response; PEG-IFN, pegylated interferon; IL, interleukin; HCV, hepatitis C virus; RVR, rapid virological response.

**Table 6:** Comparison of pretreatment factors between patients with and without 2-week virological response.

Factors	Week 2 response (≥4.56-log reduction) (*n* = 21)	Non-week-2-response (<4.56-log reduction) (*n* = 12)	*P*
Age (years)	58	62	0.618
Sex (male/female)	13/8	5/7	0.300
Height (cm)	162.8	158.3	0.291
Weight (kg)	65.8	57.5	0.175
BMI	24.7	22.0	0.593
Baseline HCV-RNA (LogIU/mL; TaqMan)	6.8	6.2	0.013
Baseline HCV-RNA (LogIU/mL; AccuGene)	6.2	5.5	0.005
Baseline HCV core Ag (fmol/L)	6134.9	1485.3	0.003
No response to prior PEG-IFN and ribavirin	1	3	0.125
Fatty liver	6	0	0.065
Cirrhosis	7	8	0.083
Diabetes mellitus	3	2	1.000
IL28B (major/minor)	15/6	6/6	0.274
Core 70 (wild/mutant)	12/8	6/6	0.718
Core 91 (wild/mutant)	15/5	8/4	0.696
WBC (/mm^3^)	5160	4435	0.175
Hb (g/dL)	14.8	14.1	0.022
Platelets (/mm^3^)	16.7	14.8	0.345
ALT (IU/L)	51.0	43.5	0.927
*γ*-GT (IU/L)	34.0	38.0	0.868
Type VI collagen 7S (ng/mL)	4.8	4.8	0.811
Hyaluronic acid (ng/mL)	94.0	105.2	0.671
AFP (ng/mL)	4.5	6.7	0.811
Reduced dose regimen	15	12	0.065

Values are expressed as the median. BMI, body mass index; HCV, hepatitis C virus; Ag, antigen; IL, interleukin; WBC, white blood cells; Hb, hemoglobin; ALT, alanine aminotransferase; *γ*-GT, *γ*-glutamyl transferase; AFP, alpha-fetoprotein.
